# Primary paraganglioma of the lung: a case report

**DOI:** 10.1186/s13256-015-0639-z

**Published:** 2015-09-02

**Authors:** Giuseppe Fiorentino, Anna Annunziata, Nicolina De Rosa

**Affiliations:** Division of Respiratory Physiopathology, Monaldi Hospital, Via Leonardo Bianchi, 1, 80131 Naples, Italy

**Keywords:** Paraganglioma, Cancer, Thoracic surgery

## Abstract

**Introduction:**

Primary paraganglioma of the lung is a rare tumor of which few cases are reported in literature. Both solitary and diffuse primary pulmonary paragangliomas are described. The solitary form of this tumor is rare.

**Case presentation:**

We report the case of a 63-year-old Caucasian man with cough, intermittent palpitations and dyspnea. A contrast-enhanced computed tomography scan of his chest revealed a rounded, high-density lesion with irregular profiles measuring 24mm in diameter in the middle lobe. The lesion was suggestive of malignancy. Fine-needle aspiration cytology was performed. The results of the cytological tests were positive for malignant cells. Surgical resection was the choice of treatment. The results of the biochemical tests and postoperative histological examination allowed a definitive diagnosis: primary pulmonary paraganglioma.

**Conclusions:**

Paragangliomas are identified and characterized with the use of computed tomography and other imaging methods, but for a definitive diagnosis, histopathological evaluation is necessary.

We report a rare case of a primary pulmonary paraganglioma that was treated surgically. This case report adds valuable knowledge to the literature on pulmonary paragangliomas.

## Introduction

Paragangliomas are rare tumors that arise from extra-adrenal chromaffin cells. They represent 10–18% of all chromaffin tissue-related tumors, which are reported at a rate of 2–8 cases/million a year [1]. Paragangliomas of the head, neck and mediastinum are usually associated with the parasympathetic system, and are chromaffin negative; they do not secrete catecholamines and do not present with episodic hypertension and other suggestive symptoms [2]. Primary pulmonary paragangliomas are rare [2]. Few cases of primary pulmonary paraganglioma have been reported since the first report by Heppleston in 1958 [3]. Erickson *et al*., in 2001, describe 28 cases of thoracic paragangliomas since 1978 (19 in the lung parenchyma). The majority (86%) of the paragangliomas below the neck were pulmonary parenchymal incidentalomas that were described as indeterminate nodules on imaging studies of the chest [4]. Pulmonary paragangliomas are difficult to differentiate from bronchial carcinomas and metastatic tumors. Imaging studies are important to confirm a clinical suspicion of paraganglioma and an important guide to design any subsequent treatment plan. However, according to the data reported in the literature, histopathological evaluation is necessary for a definitive diagnosis. The present case describes an unexpected diagnosis of a very rare cancer.

## Case presentation

**Fig. 1:**
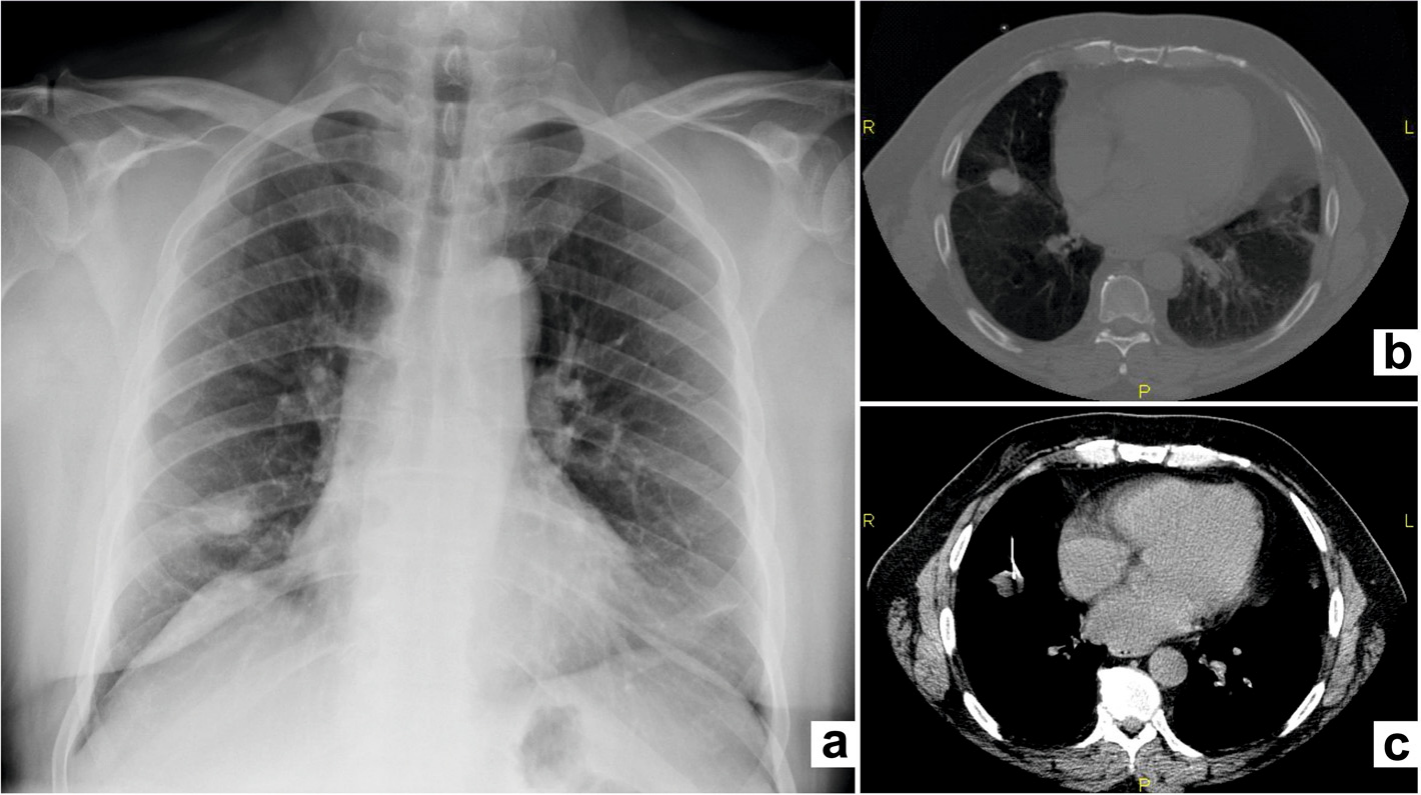
Radiological images of the pulmonary nodule. Chest X-ray showing a lesion in the right lung field (**a**). Contrast-enhanced computed tomography scan of the chest revealing a rounded, high-density lesion with irregular profiles with a diameter of 24mm in the middle lobe (**b**). Fine-needle aspiration (**c**)

We report the case of a 63-year-old Caucasian man who presented with cough, intermittent palpitations and dyspnea. Our patient had a history of smoking. His blood pressure was normal, as were the results of clinical laboratory examinations. Our patient was subjected to a respiratory functional study that showed a moderate, nonreversible obstructive syndrome (forced expiratory volume in the first second (FEV1) 58%); arterial blood gas analysis showed normal oxygen saturation. A chest X-ray showed a lesion in the right lung field (Fig. 1a).

A contrast-enhanced computed tomography (CT) scan of his chest revealed a rounded, high-density lesion with irregular profiles with a diameter of 24mm in the middle lobe, (Fig. 1b). An abdominal ultrasound scan and cervical CT scan revealed no abnormalities. The lesion was scanned by positron emission tomography (PET)/CT. The image demonstrated ^18^F-fluorodeoxyglucose (^18^F-FDG) uptake into the pulmonary lesion. Standardized uptake values (SUV) measured 2.8. Bronchofibroscopy did not show external pressure or visible alterations. Conventional and brush biopsies did not find cancer cells. Fine-needle aspiration cytology was performed (Fig. 1c). The results of the cytological tests were positive for not otherwise specified (NOS) neuroendocrine carcinoma. The immunohistochemistry test results were positive for cytokeratin (CK) AE1\AE3 and natural killer cell-associated antigen CD56, negative for thyroid transcription factor-1 (TTF-1), P63, and leukocyte common antigen (LCA). It was not possible to perform octreoscan scintigraphy due to the long waiting times envisaged.

After 30 days of therapy with long-acting beta-2 agonists, inhaled corticosteroids plus anticholinergic drugs, and a pulmonary rehabilitation cycle, the thoracic surgeon examined our patient and decided upon subsegmentectomy for histological confirmation, this type of intervention allowing a wide margin of resection free of tumor. The lesion was located in the medial segment of the middle lobe: the pulmonary hilum was surrounded by fibrous tissue that makes it difficult to isolate the vessels. Samples of pericardial fluid and serum blood were collected for cytological examination; micronodules were present, of which the largest was removed for histological examination. In the context of this fibrous tissue was also a small lymph node station 4R, which extemporaneous histological examination proved to be likely metastatic infiltration. It was removed for definitive examination.

**Fig. 2:**
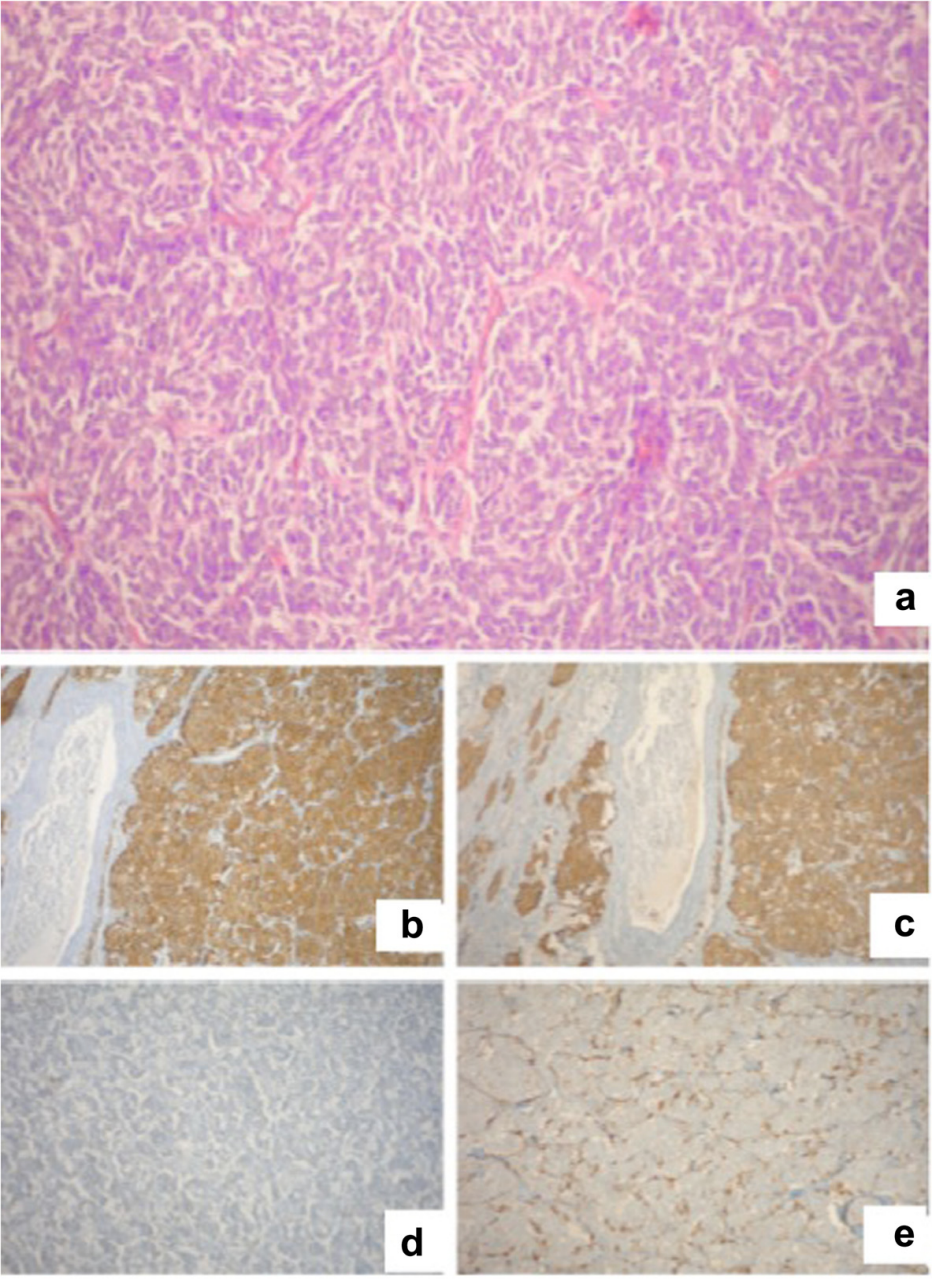
Histology of the nodule. The definitive histological examination of the nodular lesion, showed a tumor with an organoid pattern (**a**). At immunohistochemical staining, the cell population was positive for NSE, chromogranin, synaptophysin, natural killer cell-associated antigens CD56 and CD57, GFAP, and negative for CK, EMA, TTF-1 (**b**, **c**, **d**); the protein s100 has marked the base of the nests’ neoplastic cells (**e**). *CK* cytokeratin, *EMA* epithelial membrane antigen, *GFAP* glial fibrillary acid protein, *NSE* neuron-specific enolase, *TTF-1* thyroid transcription factor-1

Our patient was treated with subsegmentectomy for histological confirmation and because intraoperative frozen sections showed a malignant neoplasm, it was completed by lobectomy of the right middle lobe and complete lymphadenectomy. Extemporaneous histological examination of a pericardial micro vegetation (hyaline plaque) and lymph node station 4R (a lymph node with small parenchymal nodules composed of spindle cells and ovoid in chromatin suspected of widespread metastases) was also performed. The definitive histological examination of the nodular lesion showed a tumor with an organoid pattern (Fig. 2a).

At immunohistochemical staining, the cell population was positive for neuron-specific enolase (NSE), chromogranin, synaptophysin, natural killer cell-associated antigens CD56 and CD57, glial fibrillary acid protein (GFAP), negative for CK, epithelial membrane antigen (EMA), and TTF-1 (Fig. 2b, c, d); the protein s100 has marked the base of the nests’ neoplastic cells (Fig. 2e). The definitive diagnosis was paraganglioma of the middle lobe pT1b pN0 cM0. The lymph node was negative for malignant neoplasm; the diagnosis was small leiomyoma (+ s100-actin). In subsequent follow-ups at six months and at one year, our patient showed no problems.

## Discussion

Our patient’s medical history and the radiological studies performed showed a solitary pulmonary nodule with a risk of malignancy of 81.5% [5]. CT-guided fine-needle aspiration cytology was performed with evidence of NOS neuroendocrine carcinoma. Pulmonary aspiration as a diagnostic technique has a sensitivity of 90% and a specificity of 97% [6]. With the diagnosis of cancer suggestive of malignancy, he was examined by the thoracic surgeon. His moderate degree of obstructive syndrome was treated pharmacologically and with pulmonary rehabilitation to improve his respiratory function and to reduce the operative risk. There are no markers or radiological characteristics guidelines for the diagnosis of pulmonary paraganglioma. Unfortunately, it can be difficult to identify specific signs for this disease.

Primitive lung paragangliomas are often discovered incidentally with examination of routine chest radiographs, and patients are free of symptoms and hypertension. According to the literature about 10% of paragangliomas are malignant and all patients have been treated surgically with either local excision or lobectomy [7]. There are, however, no histological or biochemical markers that can reliably predict the malignant tendency of these tumors. The recent reclassification of neuroendocrine neoplasms of the lung shows that in this context neuroendocrine can differentiate several groups of diseases (tumorlets, carcinoids, small cell lung carcinoma, and large cell neuroendocrine carcinoma) [8].

## Conclusions

In this case report we highlight the presentation of a rare cancer as primary paraganglioma of the lung. Pulmonary paraganglioma is very similar to carcinoid neuroendocrine tumor, in fact in the preparation cytology, neuroendocrine markers were present. Often the cytology cannot allow easy differentiation of large cell neuroendocrine carcinoma from small cell cancer. Finally, only histopathological examination allowed characterization of the lesion. The biological behavior of these rare malignant neoplasms in the lung is generally favorable; however, we cannot predict the histological parameters with any certainty.

## Consent

Written informed consent was obtained from the patient for publication of this case report and any accompanying imagines. A copy of the written consent is available for review by the Editor-in-Chief of this journal.

## Abbreviations

^18^F-FDG PET: ^18^F-fluorodeoxyglucose positron emission tomography
CK: cytokeratin
CT: computed tomography
EMA: epithelial membrane antigen
FEV1: forced expiratory volume in the first second
GFAP: glial fibrillary acid protein
LCA: leukocyte common antigen
NOS: not otherwise specified
NSE: neuron-specific enolase
SUV: standardized uptake values
TTF-1: thyroid transcription factor-1
